# Supplementary material for: Structure-based mechanism for activation of the AAA+ GTPase McrB by the endonuclease McrC
Source: Nat Commun. 2019 Jul 11;10:3058. doi: 10.1038/s41467-019-11084-1 (PMC6624300; doi:10.1038/s41467-019-11084-1)
Supplement: Supplementary file 1 — Supplementary Information [file 41467_2019_11084_MOESM1_ESM.pdf]

Supplementary Information

Structure-based mechanism for activation of the AAA+ GTPase McrB by the endonuclease McrC

N. Nirwan, Y. Ithoh et al.

Supplementary Figures: 1 – 15

Supplementary Table 1 – 4

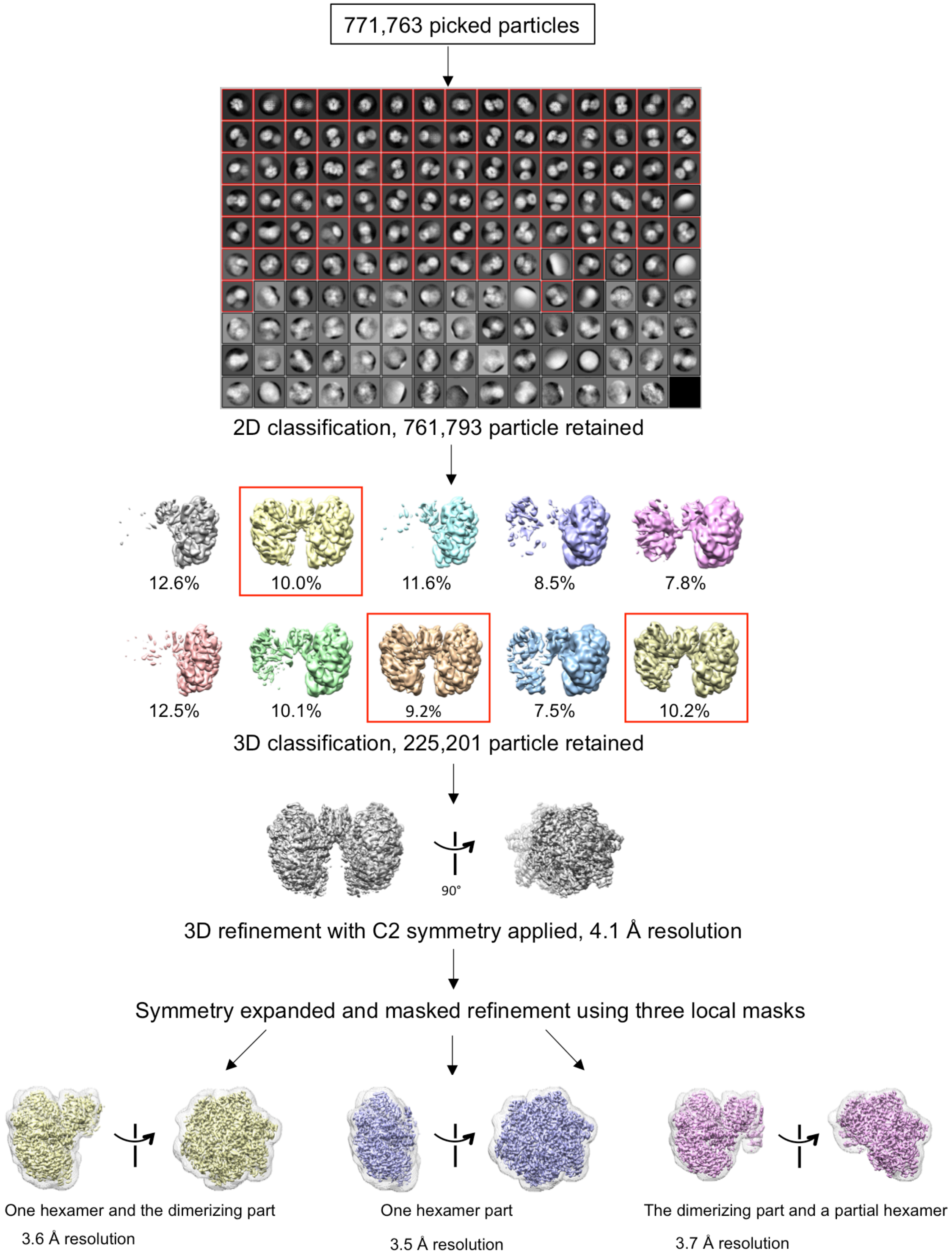

Supplementary Fig. 1. Overview of the cryo-EM data processing. Picked particles are 2D classified and the quality particles are selected after initial 3D classification. 3D refinement was performed with C2 symmetry applied. To improve local resolution, particles were symmetry expanded and refined using three masks.

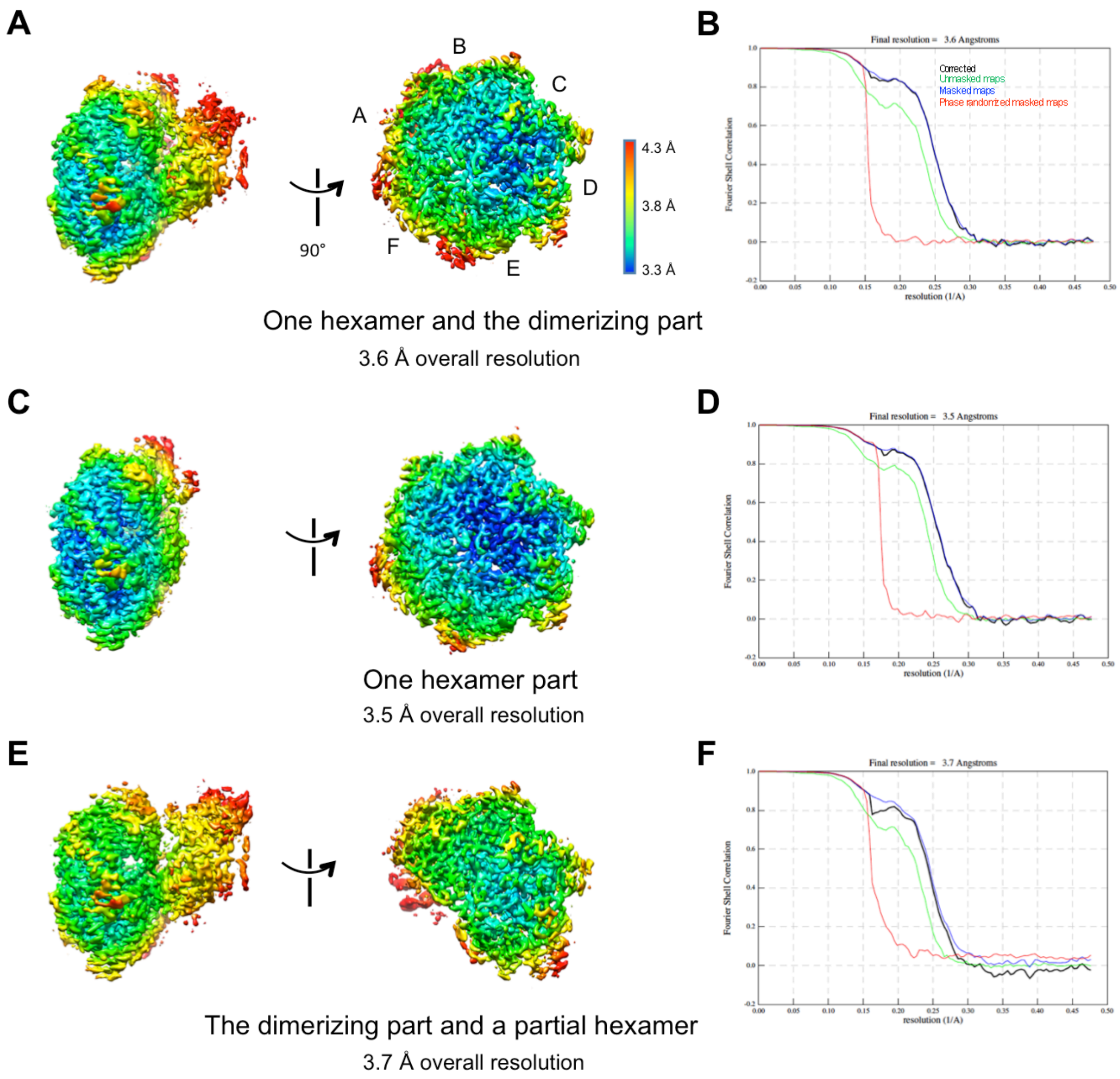

Supplementary Fig. 2. Local and overall resolution of cryo-EM maps. Local resolution of the masked cryo-EM maps was estimated by Relion and shown by colors (A, C, E). The FCS curves generated by Relion are shown (B, D, F).

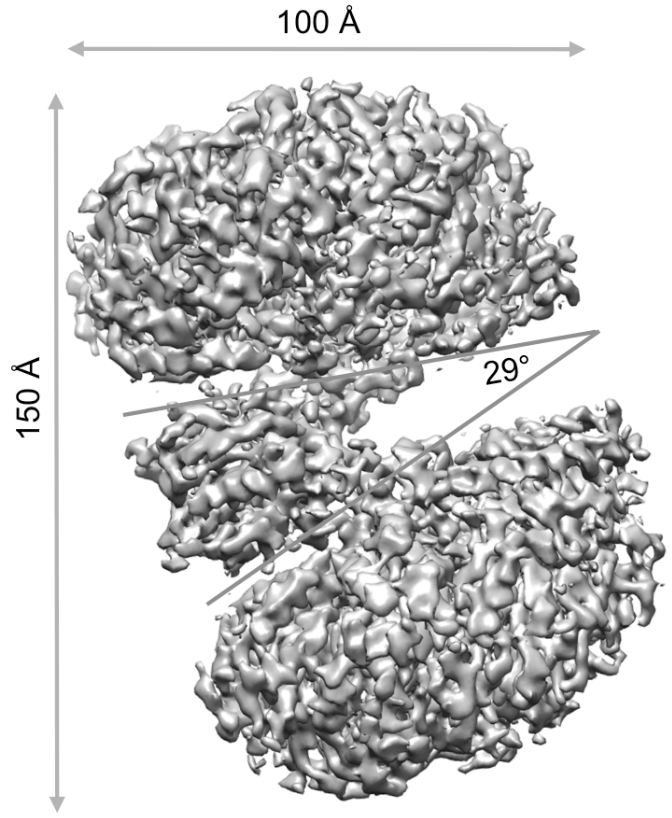

Supplementary Fig. 3. The 3.6 Å electron density map of McrBΔNC. Also shown are the approximate dimensions of the molecule and tilt angle between the two hexamers.

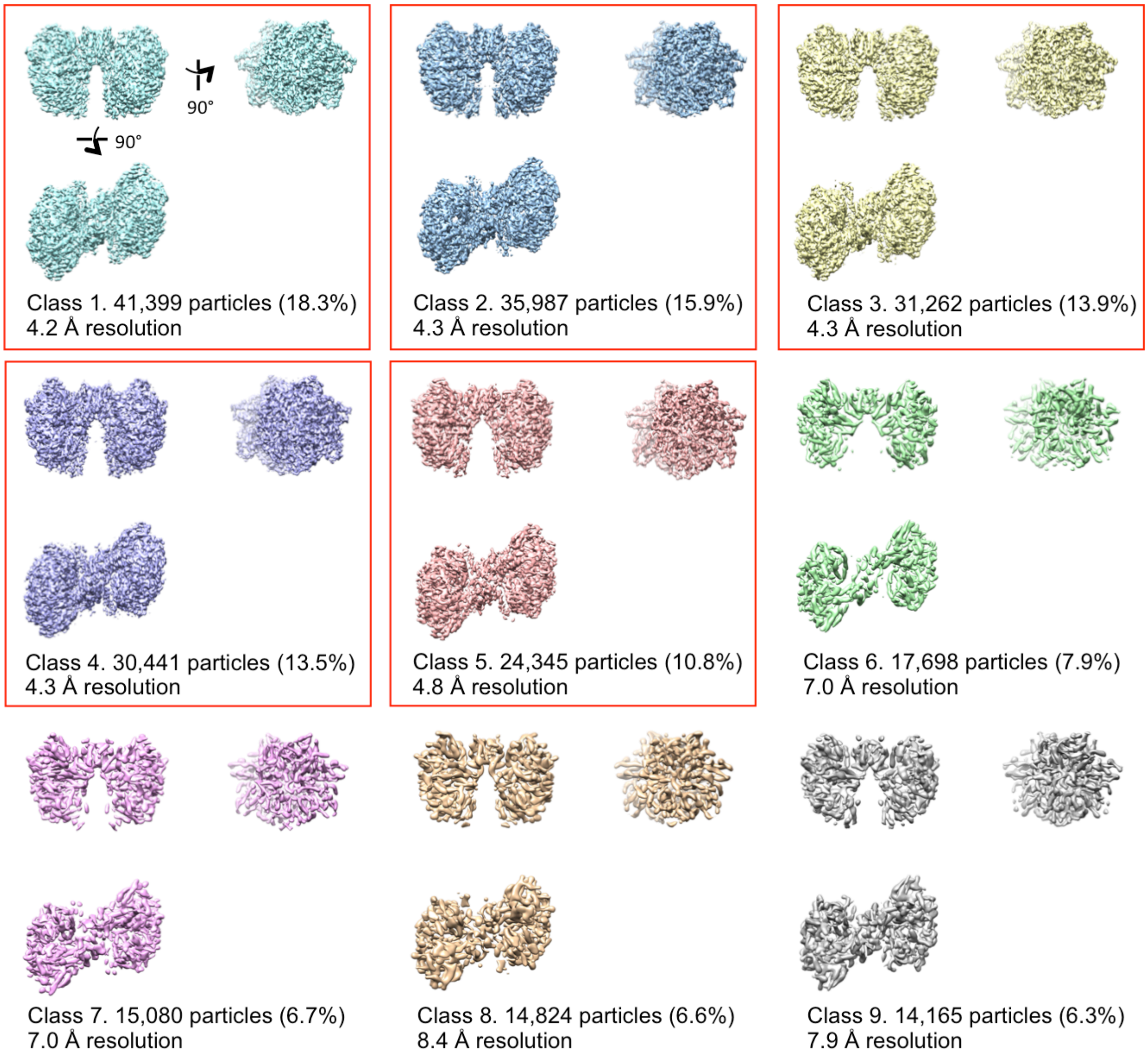

Supplementary Fig. 4. 3D classes depending on the movement of McrB hexamers. Particles are classified into nine classes by local angular search 3D classification. The particle distribution, population and resolution are shown. Five classes of them (classes 1-5) are relatively high resolution and were subjected to model building and refinement.

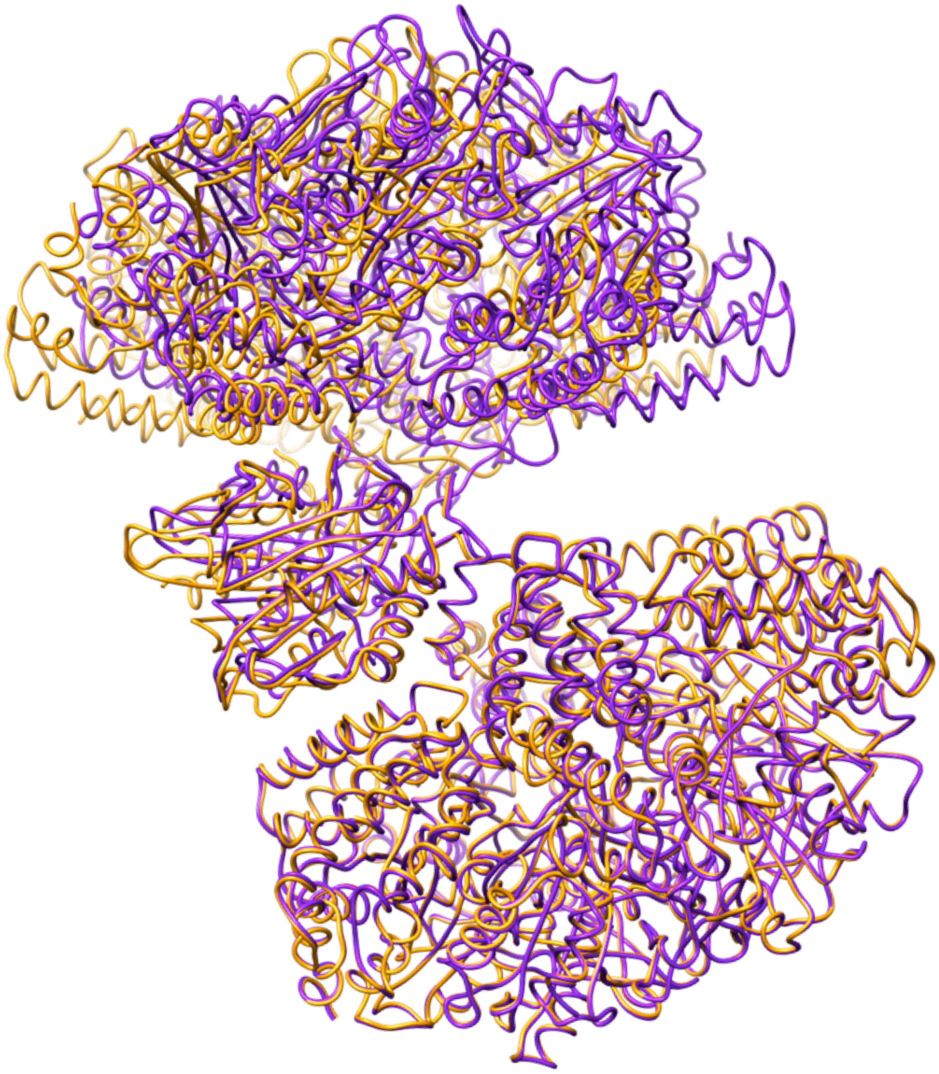

Supplementary Fig. 5. Structures of two of the nine major classes superimposed using hexamer 2 to highlight the structural plasticity of the tetradecamer.

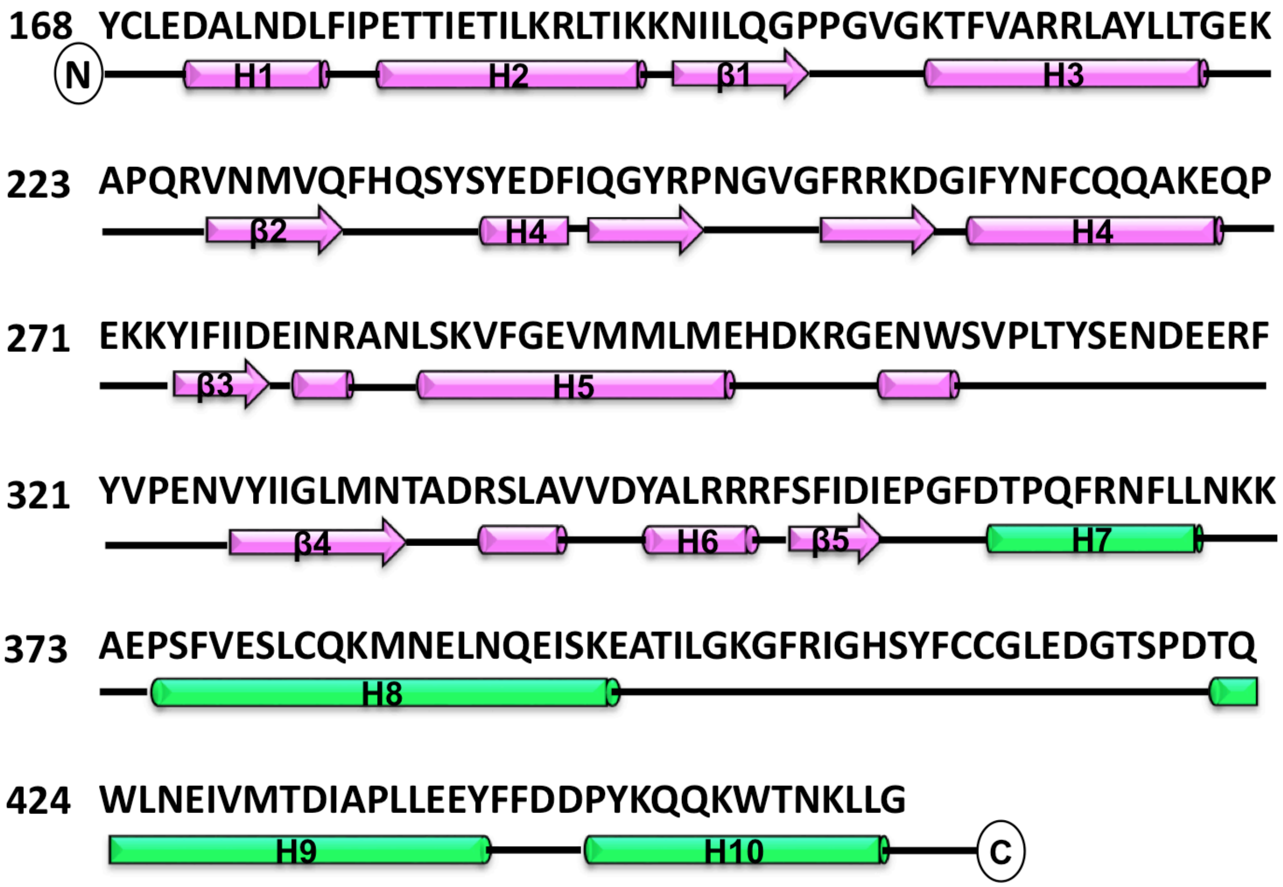

Supplementary Fig. 6. Secondary structure of McrBΔN. The helices are represented as cylinders and β-sheets are represented as arrows. The N-terminal α-β-α domain is colored in pink while the C-terminal α-helical domain is colored in green.

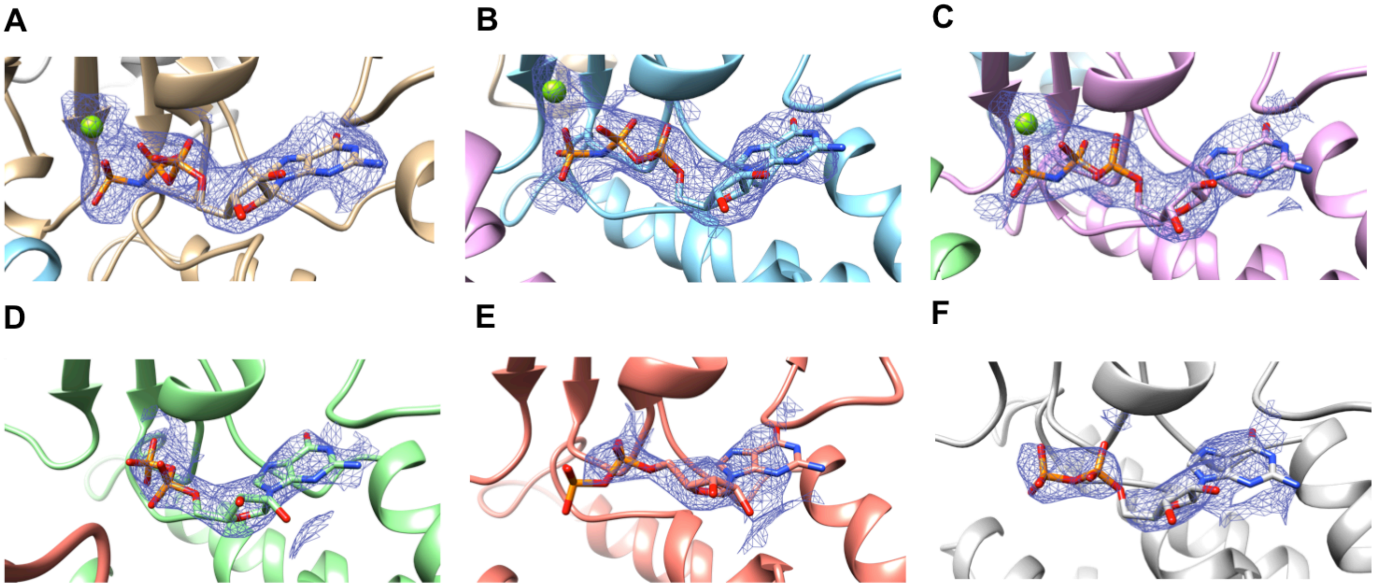

Supplementary Fig. 7. Cryo-EM density for bound nucleotides. Densities for GNP bound at A) AB, B) BC and C) CD interfaces. Densities for GDP bound at D) DE, E) EF and F) FA interfaces.

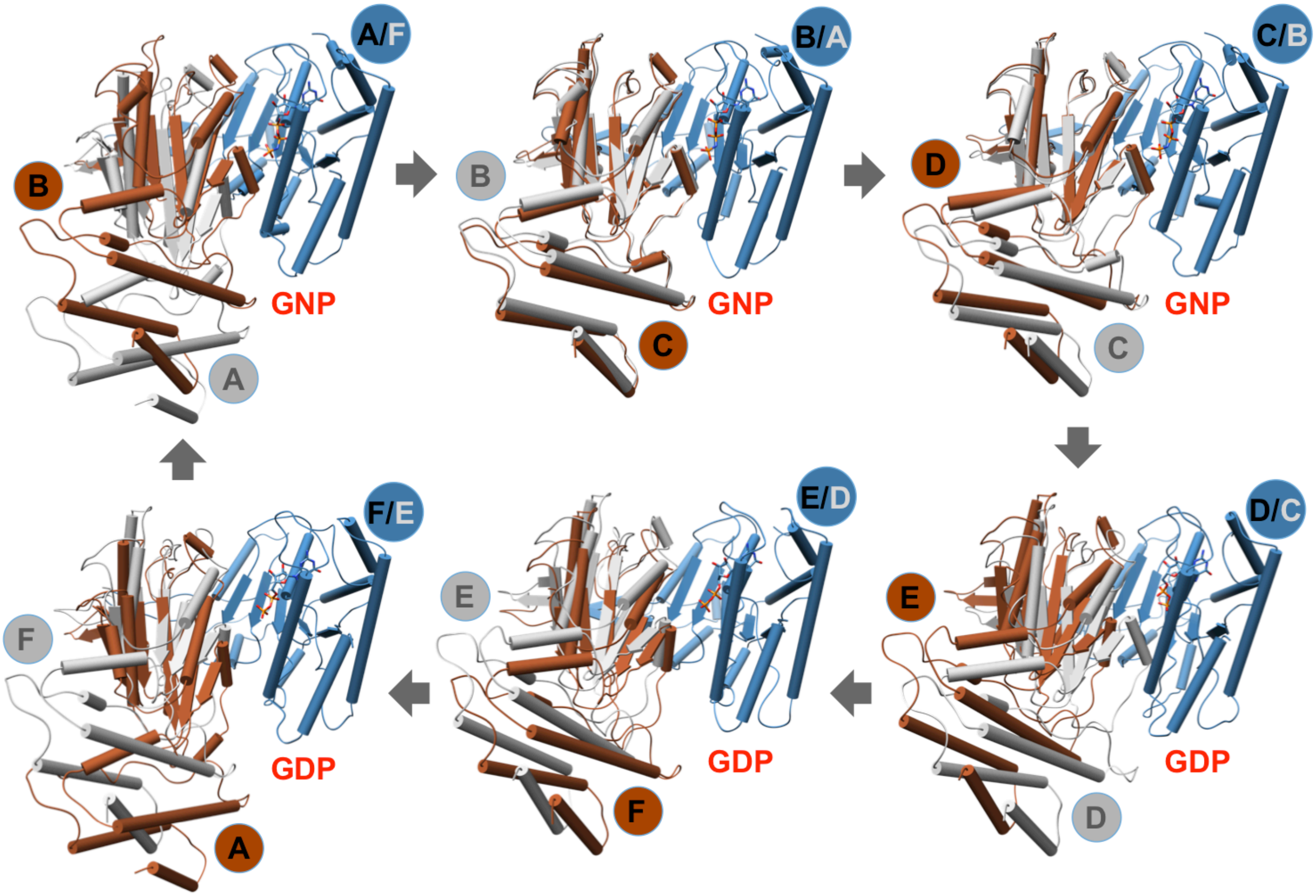

Supplementary Fig. 8. Interprotomer movement at the six nucleotide bound interfaces of McrBΔN hexamer. The figure illustrates the relative orientations of the *cis* (blue) and *trans* (brown) protomers of the nucleotide bound dimers AB, BC, CD, DE, EF and FA. The panels are arranged to follow the clockwise cyclic arrangement of the interfaces in the hexamer. The nucleotide at each interface is shown as stick. Also shown is the relative orientation of the *trans* protomer (gray) of the immediate interface in the anticlockwise direction, obtained by superimposing the NTDs of the respective *cis* protomers. For clarity only one of the *cis* protomers is shown.

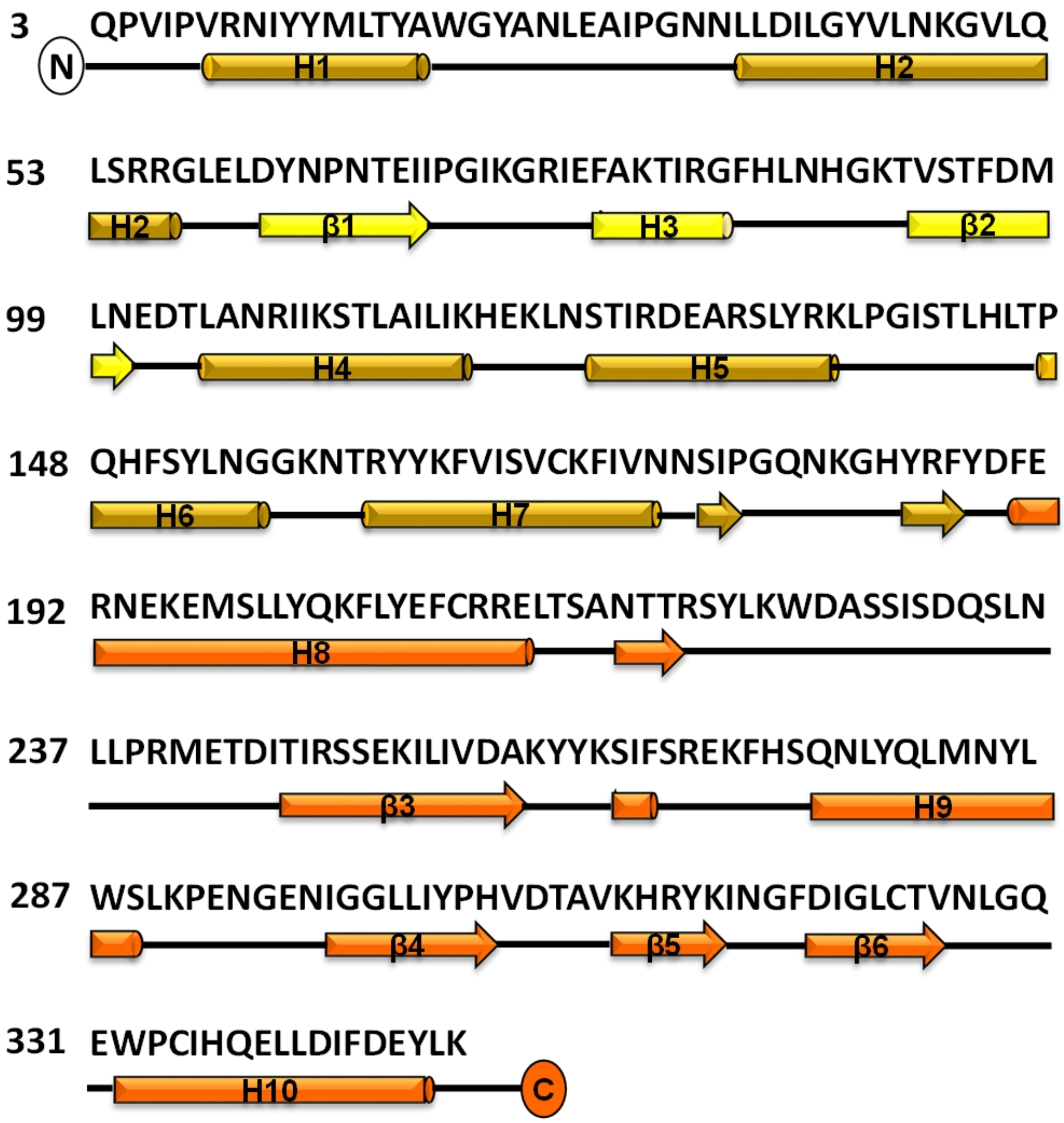

Supplementary Fig. 9. Secondary structure of McrC. The helices are represented as cylinders and β-strands are represented as arrows. N-terminal helix domain is colored in yellow, the stalk is colored in blue while the C-terminal catalytic domain is colored in orange.

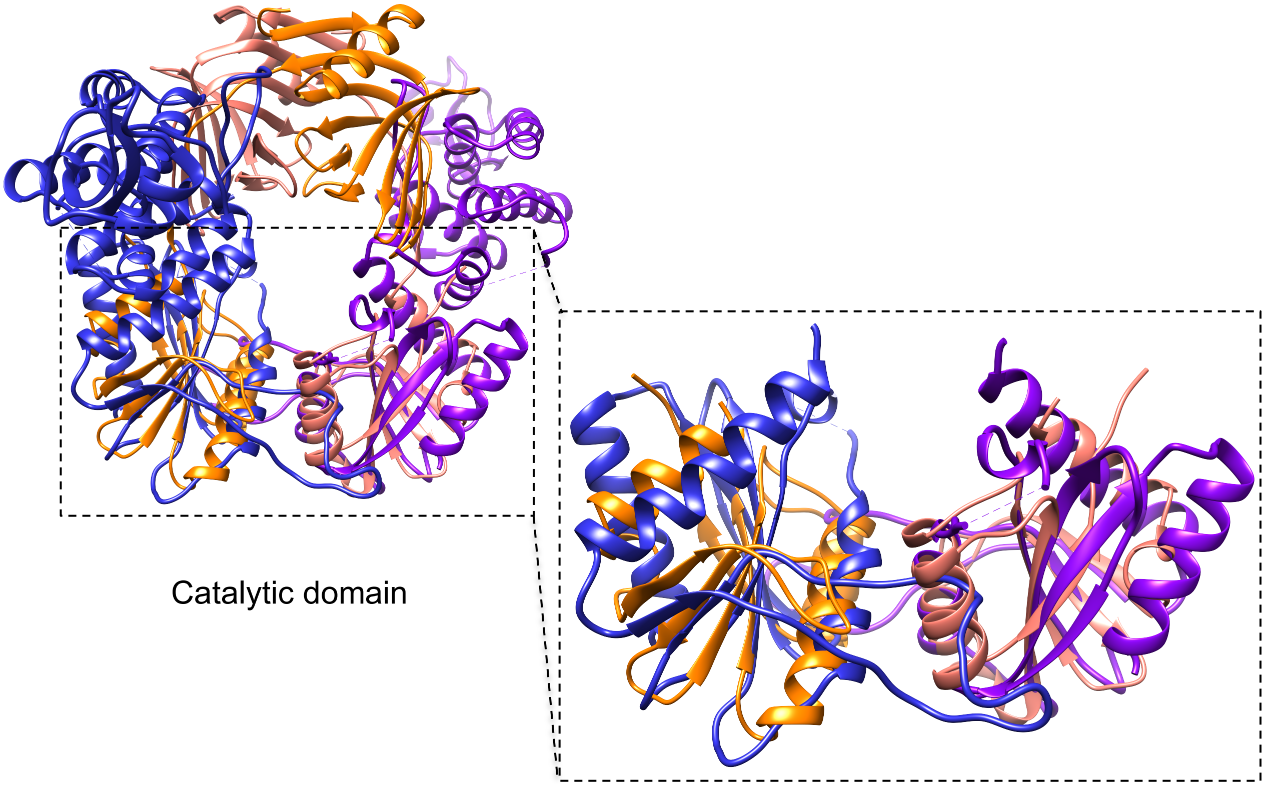

Supplementary Fig. 10. Superposition of the catalytic domains of McrC (blue and purple) and EndoMS endonuclease (orange and salmon; PDB code 5GKF). For clarity, the DNA bound to EndoMS has been hidden.

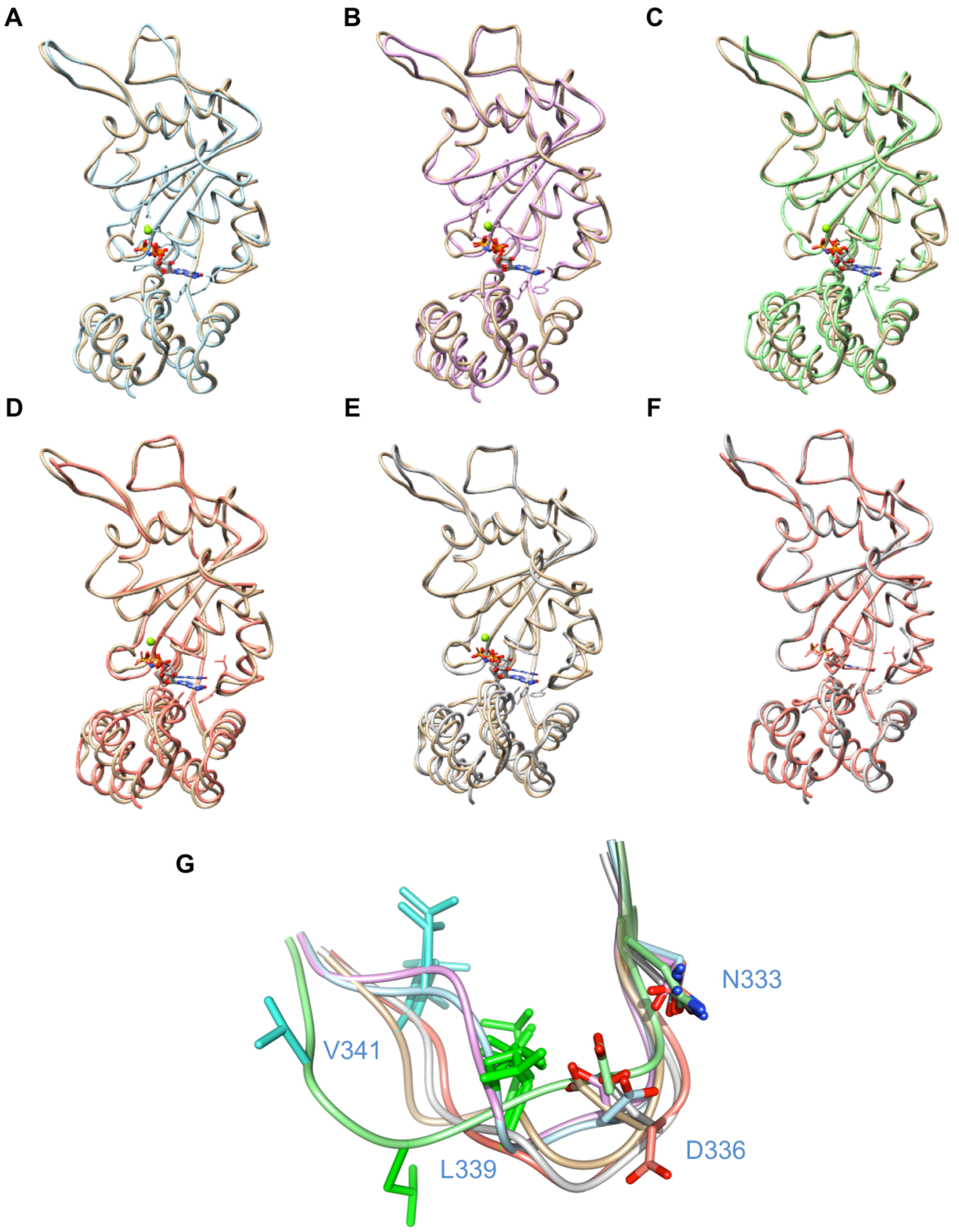

Supplementary Fig. 11. Inter-domain motion in different protomers with respect to protomer A (tan). A) Subunit B (light blue), B) subunit C (light pink), C) subunit D (light green), D) subunit E (salmon) and E) subunit F (grey). F) Superposition of subunit E on subunit F. G) Superposition of loop L3 of protomer A (tan), B (light blue), C (light pink), D (light green), E (salmon) and F(grey). Also shown are side chains of McrB-N333, McrB-D336, McrB-L339 (green) and McrB-V341 (turquoise).

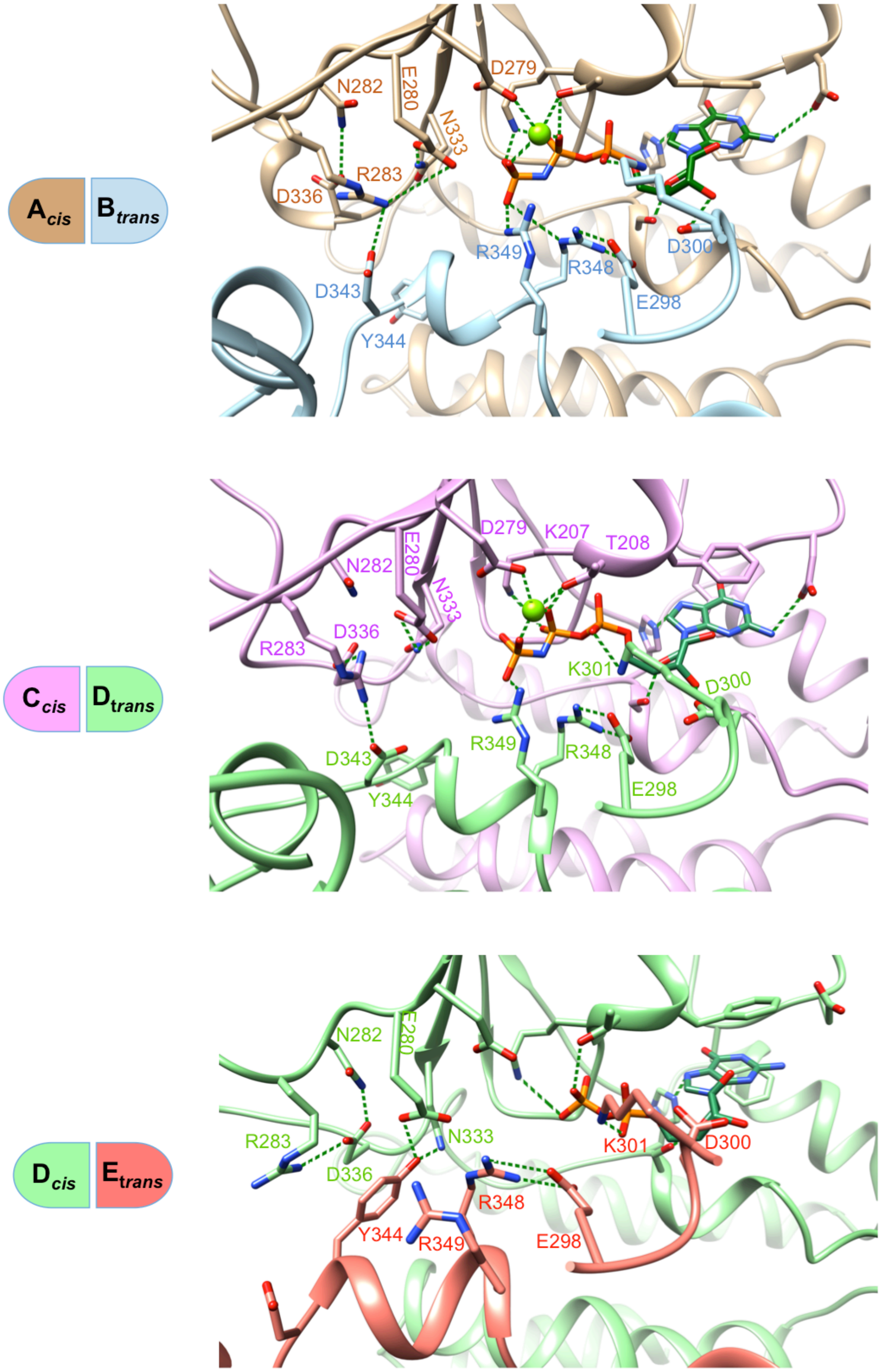

Supplementary Fig. 12. The network of interactions communicating the sensing of the γ-phosphate of GNP by McrB-Arg348 and Arg349 to McrB-Arg283 and Asp336, resulting in the rearrangement of the active site residues McrB-Glu280 and Asn333 at AB and CD interface, in comparison to the GDP-bound DE interface.

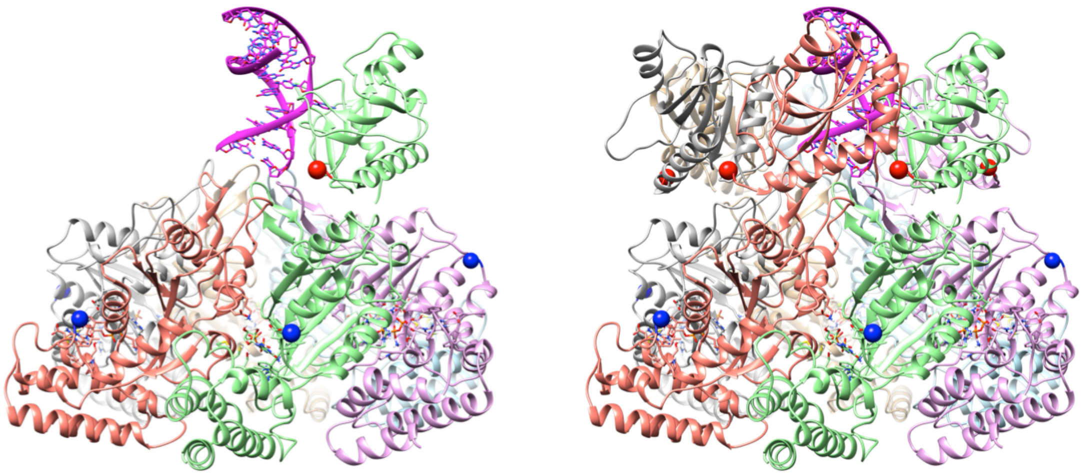

Supplementary Fig. 13. A composite model of a DNA bound N-terminal DNA binding domain (NTD) of one McrB protomer placed on the McrBΔN hexamer (left). A composite model of McrB hexamer formed of six NTDs and six AAA+ domains (right). The C-terminal end of the NTD is shown as red sphere and the N-terminal end of the AAA+ domain is shown as blue sphere to indicate the location of the linker attachment site. The linker length (not shown) connecting the two termini is 18 amino acids. The DNA is modeled to show the plausible position of the DNA bound to NTD based on the structure 3SSD[11]. In the present arrangement of the McrB AAA+ domains, based on the structure of McrBΔNC, the pore of the ring is not wide enough to allow threading of the DNA through the pore of the hexameric ring. Furthermore, the stalk of McrC would block the entry of the DNA. However, DNA could enter the pore subsequent to the rearrangement of McrB protomers (see Results).

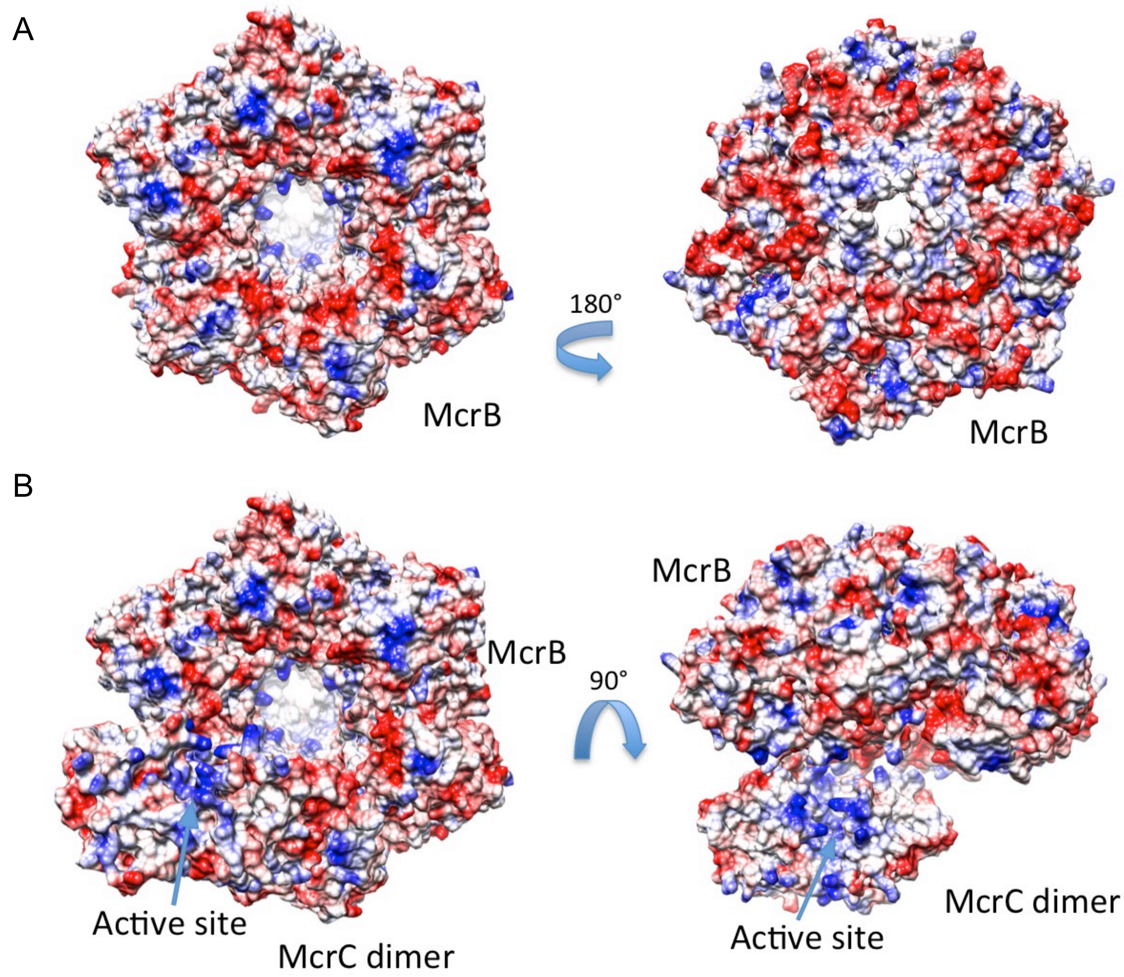

Supplementary Fig. 14. Electrostatic potential surface of McrBΔN and McrBΔNC. A) View of McrBΔN hexamer showing the face that interacts with McrC (left) and of the opposite face (right). B) View of McrBΔNC with the helical bundle and stalk of McrC removed for clarity. The active site of McrC is marked by an arrow.

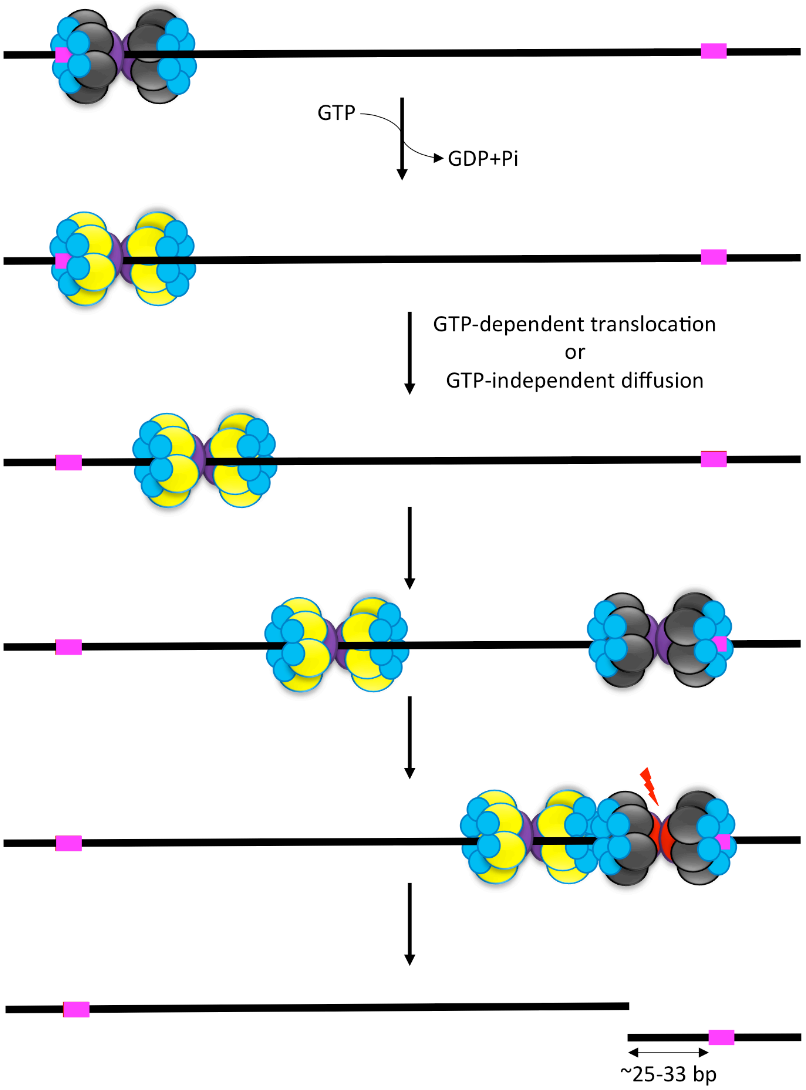

Supplementary Fig. 15: A proposed mechanism for DNA translocation/diffusion coupled DNA cleavage by McrBC. McrB hexamers (grey) assembled as a tetradecamer with a dimer of McrC (purple) on the DNA recognition sequence (magenta). The blue spots represent the DNA binding domain of McrB. A tetradecamer activated to move along the DNA is in yellow. McrC dimer activated to cleave the DNA is in red. The cleavage efficiency could be sequence dependent. It is also possible that after convergence the two tetradecamers move simultaneously away from the target site resulting in cuts further away from the site. This could explain the observation of multiple positions of cleavage observed by run off sequencing[1] or by polyacrylamide gel electrophoresis[2]. Alternatively, it is possible that one McrB hexamer is bound to a target site and another hexamer is bound to a second site, and the two are brought together by dimerization of McrC and DNA looping. DNA cleavage could result from subsequent extrusion of the looped DNA by the two hexameric McrB. Further studies are required to decipher the mechanistic details of DNA cleavage.

Supplementary Table 1. Cryo-EM data collection, refinement, and validation statistics

**Data collection**	
Microscope	Titan Krios
Camera	K2 Summit
Magnification	130,000
Voltage	300 kV
Electron dose	30 $e^-/Å^2$
Defocus range	−0.3 to −5.0 μm
Pixel size	1.05 Å
Initial particles	771,763
Final particles	225,201
**Model composition**	
Total atoms	36,381
Protein residues	2,212
Ligands (GNP/GDP/Mg^{2+})	3/3/3
**Refinement**	
Resolution	3.6 Å
Map-sharpening *B* factor	−191 $Å^2$
Model to map CC (entire box)	0.77
Model to map CC (around atoms)	0.78
Average *B* factor	
Protein	56 $Å^2$
Ligand	52 $Å^2$
RMSD bond lengths	0.009 Å
RMSD bond angles	1.0°
**Validation by MolProbity**	
Clash score	2.8
Rotamer outliers	1.2%
Ramachandran plot	
Favored	97.2%
Allowed	2.8%
Disallowed	0%
**EMDB ID**	EMD-0310
**PDB ID**	6HZ4

Supplementary Table 2. Cryo-EM data collection, refinement, and validation statistics

	**Class 1**	**Class 2**	**Class 3**	**Class 4**	**Class 5**
**Image processing**					
Resolution (Å)	4.2	4.3	4.3	4.4	4.8
Map-sharpening *B* factor (Å^2)	−181	−181	−174	−215	−214
**Model composition**					
Total atoms	67,732	67,732	67,732	67,732	67,732
Protein residues	4,120	4,120	4,120	4,120	4,120
Ligands (GNP/GDP/Mg^{2+})	6/6/6	6/6/6	6/6/6	6/6/6	6/6/6
**Refinement**					
Resolution (Å)	4.2	4.3	4.3	4.4	4.8
FSC (entire box)	0.75	0.76	0.75	0.72	0.75
FSC (around atoms)	0.76	0.77	0.76	0.73	0.76
Average *B* factor (Å^2)					
Protein	105	97	120	106	140
Ligand	106	100	124	108	144
RMSD bond lengths (Å)	0.006	0.006	0.005	0.005	0.006
RMSD bond angles (°)	0.8	0.9	1.0	0.8	1.2
**Validation by MolProbity**					
Clash score	2.7	2.8	2.7	3.1	3.1
Rotamer outliers (%)	0.70	0.59	0.59	0.59	0.51
Ramachandran plot					
Favored (%)	97.3	97.2	97.1	97.3	97.1
Allowed (%)	2.7	2.8	2.9	2.7	2.9
Disallowed (%)	0	0	0	0	0
**EMDB ID**	EMD-0311	EMD-0312	EMD-0313	EMD-0314	EMD-0315
**PDB ID**	6HZ5	6HZ6	6HZ7	6HZ8	6HZ9

Supplementary Table 3. Parameters obtained from superposition of the protomer pairs of the six McrB interfaces.

Protomer rotation	Rotation angle (º)	Polar angles of rotation (º)
A to B	58.9	102.9, 47.2, 58.9
B to C	60.5	102.8,45.5, 60.5
C to D	60.2	102.1, 44.0, 60.2
D to E	54.2	110.2, 46.0, 54.2
E to F	57.7	103.9, 42.1, 57.7
F to A	69.2	100.9, 38.9, 69.2

Supplementary Table 4. Primers used for mutagenesis

Clone	Forward primer Sequence (5' -> 3')	Reverse primer Sequence (5' -> 3')
$McrB^{E298A}$	TAATACGACTCACTATAGGG	CGTTTATCATGCGCCATTAACATCATC
$McrB^{R348A}$	TAATACGACTCACTATAGGG	GAAAATCGCGCGCGTAGGGCATAGTC
$McrB^{R349A}$	TAATACGACTCACTATAGGG	GAAAGAAAACGCTCTGCGTAGGGC

References

1. Panne, D., Raleigh, E. A. & Bickle, T. A. The McrBC endonuclease translocates DNA in a reaction dependent on GTP hydrolysis. *J. Mol. Biol.* **290**, 49-60 (1999).

2. Pieper, U., Groll, D.H., Wunsch, S., Gast, F-U., Speck, C.,Mucke, N. & Pingoud, A. The GTP-Dependent Restriction Enzyme McrBC from Escherichia coli Forms High-Molecular Mass Complexes with DNA and Produces a Cleavage Pattern with a Characteristic 10-Base Pair Repeat. *Biochemistry* **41**, 5245-5254 (2002).
