# Supplementary material for: Structure-based mechanism for activation of the AAA+ GTPase McrB by the endonuclease McrC
Source: Nat Commun. 2019 Jul 11;10:3058. doi: 10.1038/s41467-019-11084-1 (PMC6624300; doi:10.1038/s41467-019-11084-1)
Supplement: Supplementary file 3 — Description of Additional Supplementary Files [file 41467_2019_11084_MOESM3_ESM.pdf]

Description of Additional Supplementary Files

File name: Supplementary Movie 1

Description: A morph of the four major classes of McrBΔN showing the movement of the upper McrB hexamer and McrC dimer with respect to the bottom hexamer.
